# Sensorimotor Synchronization With Auditory and Visual Modalities: Behavioral and Neural Differences

**DOI:** 10.3389/fncom.2018.00053

**Published:** 2018-07-18

**Authors:** Daniel C. Comstock, Michael J. Hove, Ramesh Balasubramaniam

**Affiliations:** ^1^Cognitive and Information Sciences, University of California, Merced, Merced, CA, United States; ^2^Department of Psychological Science, Fitchburg State University, Fitchburg, MA, United States

**Keywords:** sensorimotor synchronization, timing, rhythm, visual perception, auditory perception

## Abstract

It has long been known that the auditory system is better suited to guide temporally precise behaviors like sensorimotor synchronization (SMS) than the visual system. Although this phenomenon has been studied for many years, the underlying neural and computational mechanisms remain unclear. Growing consensus suggests the existence of multiple, interacting, context-dependent systems, and that reduced precision in visuo-motor timing might be due to the way experimental tasks have been conceived. Indeed, the appropriateness of the stimulus for a given task greatly influences timing performance. In this review, we examine timing differences for sensorimotor synchronization and error correction with auditory and visual sequences, to inspect the underlying neural mechanisms that contribute to modality differences in timing. The disparity between auditory and visual timing likely relates to differences in the processing specialization between auditory and visual modalities (temporal vs. spatial). We propose this difference could offer potential explanation for the differing temporal abilities between modalities. We also offer suggestions as to how these sensory systems interface with motor and timing systems.

## Introduction

Many behavioral studies have examined human timing ability in tasks of sensorimotor synchronization (SMS) where subjects synchronize their movements to an external rhythm.

Comparisons between auditory metronomes and visual flashing metronomes reveal that movement synchronization is less variable and can occur at faster rates with auditory metronomes (Chen et al., 2002; Repp, 2003; Repp and Penel, 2004; Lorås et al., 2012). However, visuo-motor synchronization greatly improves when synchronizing with a *moving* periodic visual metronome (Hove et al., 2010). Adding a changing velocity profile to the moving visual metronome further reduces variability in SMS tapping (Hove et al., 2013a; Iversen et al., 2015), and Gan et al. (2015) suggests that a more realistic velocity profile can bring visual SMS to be as temporally precise as auditory SMS, at moderate but not fast tempi. While most studies of SMS look at finger tapping, others have included synchronized circle drawing, gait, dancing, and eye movements in the context of modality-specific timing effects (e.g., Repp and Su, 2013).

Studies on auditory and visual interference also suggest auditory timing is more prominent. When concurrent auditory metronomes and visual flashing metronomes are presented out-of-phase, the auditory sequences interfere with visuomotor timing, but not vice versa (Chen et al., 2002; Repp and Penel, 2002, 2004). The interference effect is considerably reduced with moving visual metronomes and is tied to training and experience as the auditory dominance is stronger in musicians and weaker in video gamers (Hove et al., 2013a). Similarly, auditory cues can improve visual temporal discrimination (Morein-Zamir et al., 2003; Parise and Spence, 2008). This effect only holds for the temporal domain however, as the visual system dominates when auditory and visual stimuli conflict in the spatial domain; spatial dominance in the visual modality is apparent in the well-known “ventriloquist effect” (Vroomen et al., 2001).

## Role of error correction in timing

Error correction is a crucial component of any SMS task. By inducing perturbations and errors in SMS, we can gain insight into the underlying timing mechanisms. A common method to induce errors in a SMS task is to occasionally perturb an otherwise isochronous metronome (Repp, 2000, 2001a,b; Praamstra et al., 2003; Repp and Keller, 2004; Jang et al., 2016; Jantzen et al., 2018). Error correction in SMS can be broken down into two distinct mechanisms: a phase-correction mechanism for correcting errors in relative phase, and a period-correction mechanism that corrects changes to the internal timekeeper period (Repp, 2001b; Repp and Keller, 2004). Period corrections require conscious awareness of the error as it involves a conscious updating of the internal rhythm; while a phase correction can happen even with errors too small for conscious awareness and does not involve updating the central timekeeper period and so is considered a more peripheral process than period correction (Repp, 2001b, 2005). An error corrected under the phase-correction mechanism is typically a gradual adjustment that occurs over several beats, while an error corrected under the period-correction mechanism will be evidenced by a pronounced correction, usually followed by a more gradual phase-correction-like pattern after the initial large correction (Repp, 2001b).

While error correction has been well documented in auditory SMS, relatively little work has investigated error correction in visual SMS. In a recent study comparing error correction for auditory and flashing visual sequences, we observed error corrections for perturbations in the auditory condition that were modulated by the direction of the perturbations, but no such modulation was found for perturbations in the visual condition (Comstock and Balasubramaniam, 2017a). This suggests the visual system may not engage in the same SMS timing mechanisms as the auditory system. Additional evidence for a discrepancy in error correction for auditory and visual sequences can be gleaned from the autocorrelation structure of adjacent taps: unlike auditory SMS, tapping with visual flashes does not produce a negative lag1 autocorrelation that can indicate of the presence of a robust central timekeeping and error-correction mechanism (Hove and Keller, 2010). However, visuomotor synchronization with moving and apparent-motion metronomes do produce a negative lag1 autocorrelation, suggesting that a moving visual metronome may engage error correction (Hove and Keller, 2010; Hove et al., 2010); note that negative lag1 autocorrelation does not necessarily stem from error correction and can arise from other timing factors (e.g., Wing and Kristofferson, 1973). It remains unclear if error correction will occur with perturbations in moving visual metronomes or with larger phase perturbations in a flashing visual metronome.

## Underlying physiology of the auditory and visual timing system

### Brain networks involved in timing activity

Investigating the neural underpinnings in auditory and visual timing is a massive undertaking due to the many different timing subprocesses and tasks, including: SMS, interval timing, rhythm perception, timing recall, time perception, etc. Excellent reviews of the brain mechanisms involved in various timing activities include: a review of neural activity in music production (Zatorre et al., 2007); a review of neural activity involved in time perception (Wiener et al., 2010); and an overview of neural activation in SMS as part of a larger review of SMS (Repp and Su, 2013). This body of work consistently demonstrates that temporal processing across tasks and sensory modalities relies heavily on the motor system. This motor network includes the supplemental motor area (SMA), primary motor cortex, lateral premotor cortex, anterior cingulate, basal ganglia, and cerebellum (Repp and Su, 2013). Auditory rhythm perception activates the motor system and is closely linked to movement (Janata et al., 2012; Iversen and Balasubramaniam, 2016; Ross et al., 2016a,b). The SMA is also strongly implicated in motor timing (Coull et al., 2016; Merchant and Yarrow, 2016), and along with the pre-SMA could be a hub of motor timing (Schwartze et al., 2012). Subcortical regions are especially active during sub-second time perception (Wiener et al., 2010), sub-second interval timing (Repp and Su, 2013), and rhythm timing (Grahn and Rowe, 2009; Wiener et al., 2010; Coull et al., 2011; Teki et al., 2011; Hove et al., 2013b). There is evidence of a dorsal auditory stream connecting the auditory cortex to the motor cortex through the posterior parietal cortex that plays a role in rhythm perception (Patel and Iversen, 2014; Ross et al., 2018). Interestingly this dorsal stream is also implicated in visual and tactile rhythm perception (Araneda et al., 2017; Rauschecker, 2017), adding to the idea of a common timing system tied to the motor system. Further evidence of the common timing system is found in a study of auditory and visual synchronization that dissociated modality and tapping stability –putamen activation was highest when synchronizing to auditory beeps, moderate with a frequency-modulated siren and with a moving visual metronome, and lowest with a flashing visual metronome, closely paralleling behavioral performance (Hove et al., 2013b).

While visual SMS activates many of the same motor regions as auditory SMS (Hove et al., 2013b; Araneda et al., 2017), some activations are specific to the visual system. The visual cortex shows activity related to interval timing that follows the expected scalar property, such that size of timing errors measured in the visual cortex scale in proportion to size of the interval being timed as predicted by Weber's law (Shuler, 2016). Additionally, Zhou et al. (2014) found evidence that visual feature processing in the early visual cortex can contribute to duration perception, furthering the notion that at least some timing information is processed independently within the visual cortex. Additionally, in visual rhythm perception, the visual cortex plays a role predicting rhythmic onsets (Comstock and Balasubramaniam, 2017b, 2018). The additional activations with visual timing tasks, taken together with behavioral results, suggest the timing accuracy in visual processing may be compared to the auditory system due to the additional computational demands of processing the higher complexity of visual spatial information along with temporal information.

### Role of cortical oscillations in timing encoding and spreading information across the brain

In addition to looking at the networks and regions involved in temporal processing, a growing body of work shows the role of cortical oscillations in encoding timing across multiple frequency bands. Cortical oscillations play a role in connecting regions across the brain, with higher frequencies utilized for localized interaction and lower frequencies for longer range interaction (Sarnthein et al., 1998; Von Stein and Sarnthein, 2000). This pattern of oscillations is used to connect and calibrate disparate timing systems in the brain (Gupta and Chen, 2016). Oscillations relating to timing appear to arise from multiple context-specifc timing systems in the brain (Wiener and Kanai, 2016). The question is then how these functionally and anatomically disparate systems integrate and interact. It appears that oscillations from different timing systems are coordinated within the striatum (Matell and Meck, 2004; Gu et al., 2015).

Beta band activity (~20 Hz) is tied to the motor system and several studies indicate beta's role in predicting timing of auditory rhythms (Fujioka et al., 2009, 2012, 2015). Additionally, beta activity reflects top-down imposition of metrical structure on auditory rhythms (Iversen et al., 2009). Recently, beta activity has also been linked to timing predictions within the visual system in response to visual rhythms (Comstock and Balasubramaniam, 2017b).

With rhythm perception, evidence shows that internal oscillations arise to match the fundamental frequency of the rhythm, and frequency of the meter (Nozaradan et al., 2011), as well as to the frequency of imagined rhythms (Okawa et al., 2017). These findings align with the Neural Resonance Theory that posits neural rhythms synchronize to auditory rhythms, and these neural rhythms can influence attention, expectancy, and motor planning (Large and Snyder, 2009). As of yet, it is unclear if this same neural resonance to meter would arise with visual stimuli.

### Neural underpinnings of error correction

The neural correlates of error correction reveal more evidence for multiple interacting and overlapping timing mechanisms. Error detection of timing perturbations in auditory SMS tasks modulates the P1, N1, and N2 auditory ERP components depending on both the size and direction of the perturbation (Praamstra et al., 2003; Jang et al., 2016). Jantzen et al. (2018) also found a theta response stemming from the Pre-SMA and anterior cingulate for error detection, an increase in theta coupling between the SMA and the motor cortex for late perturbations. In visual error detection, the visual P1 component is reduced in latency only for large late perturbations (Comstock and Balasubramaniam, 2017a). Each of these instances show cortical activation specific to a type of perturbation, although these effects are generally limited to larger perturbations.

Smaller perturbations that elicit a phase-correction response are believed to be driven primarily by subcortical mechanisms. Applying repetitive TMS to downregulate motor and premotor cortices produced no effect on phase correction (Doumas et al., 2005), whereas phase-correction was impaired by repetitive TMS to the cerebellum (Bijsterbosch et al., 2011). This fits with the suggestion that phase-correction is primarily subcortical based on evidence from how rapidly the movement trajectory changes after a perturbation (Hove et al., 2014). A possible network that exhibits the rapid timing required for the phase-correction response is a cortico-striatal circuit connecting the cerebellum to the SMA-striatal network via the thalamus (Kotz et al., 2016).

The data on the neural underpinnings of error correction suggest multiple timing systems, each with specific roles, yet able to coordinate for rapid response. Commensurate with this idea is work suggesting the basal ganglia integrates various timing systems through oscillation comparators (Matell and Meck, 2004; Gu et al., 2015). The limited data on visual error correction, however, leave open how well this network can interface with the visual timing systems.

## Evidence the auditory system has privileged access to timing systems

Considering the auditory system's timing advantage along with the prominence of the motor system in timing processing, we suggest that the auditory system's advantage in timing stems from its stronger coupling to the motor system. Auditory timing compared to visual timing tasks often yield more activation in motor structures, such as the SMA and premotor cortex (Jäncke et al., 2000). Even when visual SMS tasks employed the modality-appropriate *moving* visual metronomes, audio-motor synchronization with auditory beeps yielded greater activation in the putamen (Hove et al., 2013b). Likewise, priming a visual rhythm with a similar auditory rhythm resulted in increased putamen activation compared to a visual rhythm alone, while a visual rhythm yielded no priming effect on an auditory rhythm (Grahn et al., 2011). The finding that the increased visual synchronization ability provided by a bouncing ball does not transfer to purely perceptual rhythm perception provides further evidence of the role of motor coupling in timing tasks (Silva and Castro, 2016). Additionally, the privileged link between auditory and motor systems can be seen in Parkinson's disease, a disorder that impairs movement due to cell loss within the basal ganglia (Davie, 2008). For example, Parkinsonian gait can improve when cued by an external rhythm, and these interventions are more effective when synchronizing with auditory metronomes than with flashing visual metronomes (Rochester et al., 2005; Arias and Cudeiro, 2008).

Visual timing activities recruit timing centers within the visual system that, based on behavioral results, are less precise compared to the auditory timing system. In Jäncke et al. (2000), visual timing tasks resulted in increased activity in the right superior cerebellum, vermis, and right inferior parietal lobe compared to auditory timing tasks. Visual timing tasks also recruit areas MT, V5, and the superior parietal lobe, tying into the dorsal visual stream (Jantzen et al., 2005), and visual rhythm perception induces increased beta activity at event onsets arising from the visual cortex (Comstock and Balasubramaniam, 2017b). It is unclear if these timing activations in the visual system are the result of compensating for a weaker connection to the motor timing system. It may be that the temporal processing in the visual system is additional processing of visual information required to interface with the motor system.

While differences in coupling strength to the motor system are crucial for modality timing differences, other factors are likely. To that end, it is clear that the visual system is able to pick out high speed temporal information, for example, V1 will phase lock its input/output to up to a 100 Hz visual flashing stimuli (Williams et al., 2004). This suggest that entrainment is not easily transferred to the systems involved in time/rhythm perception, especially at the time frame usually involved in rhythm perception, indicating that the issue may be one of translation. A likely place for that translation would be within the dorsal pathway, which has been found to have neurons with high temporal resolution in macaques, with higher temporal resolution in the auditory dorsal stream (Rauschecker, 2017). If there is a higher temporal resolution of the auditory dorsal stream than in the visual dorsal stream, then it may give explanation as to why the visual system cannot synchronize at the higher frequencies achieved by the auditory system. Of course, it cannot be ruled out that the difference in temporal resolution is due to different levels of timing precision available to the dorsal stream. Reduced timing precision in the visual stream may be caused by increased necessary processing due to richer sensory input of the visual system compared to the auditory system. Indeed, greater processing requirements and longer processing time may help to account for the inability of the visual system to allow for synchronization at the higher tempos allowed by the auditory system.

## Role of the vestibular-tactile-somatosensory system

Another link between auditory and motor systems is that auditory rhythm perception may be tied to the vestibular-tactile-somatosensory (VTS) system, which is important for movement and dance, and therefore closely tied to the motor system and attuned to timing (Todd and Lee, 2015). In addition to its ties for movement, the VTS system is clearly tied to the auditory system with regards to rhythm perception (Phillips-Silver and Trainor, 2005, 2007, 2008; Trainor et al., 2009), and through common neural activation (Araneda et al., 2017). These ties between the auditory and VTS system may be an additional factor in the dominance of the auditory system in the temporal domain.

Since VTS rhythms are ubiquitous in fetal life through the mother's gait, heart rate, breathing, etc., and since these networks are tied into auditory rhythm systems, it is likely that the VTS system is heavily tied into the timing systems used in auditory rhythm perception and in motor rhythm production (Provasi et al., 2014). This is further strengthened by the fact that movement and rhythms are linked and proprioception (part of the VTS system) plays a large role in perception of rhythms that is tied into auditory rhythm perception and production (Trainor et al., 2009). Interactions between the VTS system with visual rhythm perception remains mostly unexplored at this point however, so it is unclear how much this system plays a supramodal role in the timing involved in rhythm perception/production, or if it is only tied to the auditory and motor rhythm timing systems. Further research in this area is needed to answer these questions.

## Evolutionary origins of sensorimotor synchronization

In an evolutionary context, it makes sense that auditory and motor systems would be tightly interconnected. First, rhythms in language are critical for both perception and production and may be a driver of SMS ability (Patel, 2006). Beyond language, matching movement to sound is a necessary result of human evolution that allows for the social and cultural inclination of humanity via music (Hagen and Bryant, 2003; Brown and Jordania, 2013). Dance is also tightly connected with music and culture and can provide a further explanatory account of human SMS capability and the connection between the motor and auditory systems (Fitch, 2016; Iversen, 2016; Laland et al., 2016; Ravignani and Cook, 2016).

Beyond humans, common adaptations appear to increase SMS ability in several non-human species capable of some level of audio-motor entrainment such as parrots (Patel et al., 2009), bonobos (Large and Gray, 2015), and sea-lions (Cook et al., 2013). Although some animals can exhibit rhythmic capabilities, some remarkably well like Ronan the sea-lion (Rouse et al., 2016), they are in some ways limited compared to humans (Patel and Iversen, 2014; Merker et al., 2015). Even though there are animals that can entrain to auditory rhythms, only humans appear to be naturally inclined to do so (Wilson and Cook, 2016). Finally, there is some evidence that non-human primates are able to synchronize their movements to predictable visual stimuli (Takeya et al., 2017), yet there has been much less research on visual SMS compared to auditory SMS in non-humans.

## General synthesis and future directions

In looking at how the brain processes timing information, it is clear that many context sensitive mechanisms interact and coordinate to provide optimal timing output. Much of this interaction appears to happen within the motor system and likely involves the subcortical systems to coordinate the various mechanisms. Current research suggests that oscillations play a key role coordinating the interactions among various timing circuits. However, it is not clear if the various timing systems compute measures of time in the same way. When considering that auditory and visual systems take in very different kinds of information and use it in different ways, i.e., auditory has a stronger temporal precision, and visual has a strong spatial bias, it seems likely that the timing mechanisms themselves may greatly differ.

Consider the difference between extracting timing information between a moving visual rhythm and an auditory rhythm. Moving visual stimuli contain more information than auditory stimuli, such that while entraining to auditory stimuli, prediction of the onset of the next event involves encoding the interval between two events and utilizing that information to predict the onset of the next event. With a moving visual rhythmic stimulus, that interval information is present, but so is information on position/velocity/acceleration. This means predictions of the onset of the next event can be made as part of a continuous process. The fact that even with this information, visual SMS is at best equal to auditory SMS except at fast speeds, begs the question as to why visual SMS is less capable. One possible explanation for this is that the visual system has to encode much more information, and further, encoding that information into a form that is usable by the motor network may require extra processing. This may explain the timing activity found within the visual cortex during visual SMS. Even when there is a simple flashing metronome, there is a measure of timing activity originating from the visual cortex. Considering the reduced temporal ability with visual flashing metronomes, it suggests there may be a translation issue in harnessing a system not optimized to temporal processing the way the auditory system has been, resulting in a weaker connection to the motor timing network.

Different timing systems likely employ varying mechanisms and computational principles that are appropriate to the time scale, cellular properties, and general needs of the system. Existing computational models that capture a range of these phenomena across levels include: pacemaker accumulator models, multiple oscillator models, memory trace models, random process models, ramping activity models, delay line models, and state space trajectory-based models (Addyman et al., 2016; Hass and Durstewitz, 2016). Such models help illustrate the variety of ways to process timing information within a neural network. Evidence also suggests that cells with specific timing mechanisms exist in the basal ganglia and cerebellum (Lusk et al., 2016), yet other areas with multiple functional properties also process timing, such as in the prefrontal cortex (Hyman et al., 2012) and hippocampus (MacDonald et al., 2011). The areas that have multiple functions, as in the hippocampus and prefrontal cortex, will then likely have different computational approach than more specialized timing structures.

Given that there are multiple ways to process timing, and that many forms of cognition require some form of temporal processing, it would be surprising to find that timing mechanisms are not ubiquitous in the brain. This raises an important question. If many different timing mechanisms are available for a given task, and only one output (through action), how do neural systems arrive at the best timing information to use? A strong candidate explanation for this would implicate a mechanism that helps integration through an optimal Bayesian process (Hass and Durstewitz, 2016). Evidence from multimodal sensory integration suggests that when timing information is presented from multiple modalities, the modalities are combined and weighted based on reliability in Bayesian optimal solution (Ernst and Banks, 2002). Since most timing related activity requires motor output, we would expect that the source of timing to be utilized would be determined before, or as that timing information becomes available to the motor system. This seems to make the case that the striatal cells operating as a comparator may be the seat of the Bayesian process to determine the optimal timing source for motor timing.

Since there is some disparity in the amount of work on auditory and visual SMS error correction, there is a need to further study the error correction capabilities within visual SMS. It is currently unknown if visual error correction can be as fast as auditory error correction when dealing modality appropriate stimuli, such as a moving visual sequence or bouncing ball. Another major area of needed work is in understanding the mechanisms by which the Bayesian optimal timing source is chosen in cases where multiple sources are available. If timing mechanisms are as ubiquitous in the brain as evidence suggests, then there may be a variety of ways these mechanisms interface with the motor timing system to produce a single output. Further imaging and computational work is required to understanding this mechanism.

## Author contributions

All authors listed have made a substantial, direct and intellectual contribution to the work, and approved it for publication.

### Conflict of interest statement

The authors declare that the research was conducted in the absence of any commercial or financial relationships that could be construed as a potential conflict of interest.
